# *Piper auritum* ethanol extract is a potent antimutagen against food-borne aromatic amines: mechanisms of action and chemical composition

**DOI:** 10.1093/mutage/geae011

**Published:** 2024-03-23

**Authors:** Sandra L Hernández-Ojeda, Javier Jesús Espinosa-Aguirre, Rafael Camacho-Carranza, Jessica Amacosta-Castillo, Ricardo Cárdenas-Ávila

**Affiliations:** Instituto de Investigaciones Biomédicas, Universidad Nacional Autónoma de México, Tercer Circuito Exterior sin Número, Ciudad Universitaria, Ciudad de México 04510, Mexico; Instituto de Investigaciones Biomédicas, Universidad Nacional Autónoma de México, Tercer Circuito Exterior sin Número, Ciudad Universitaria, Ciudad de México 04510, Mexico; Instituto de Investigaciones Biomédicas, Universidad Nacional Autónoma de México, Tercer Circuito Exterior sin Número, Ciudad Universitaria, Ciudad de México 04510, Mexico; Unidad de Servicio de Apoyo a la Investigación y a la Industria (USAII), Facultad de Química, Universidad Nacional Autónoma de México, Avenida Universidad 3000, Ciudad de México 04510, Mexico; Facultad de Ciencias, Universidad Nacional Autónoma de México, Avenida Universidad 3000, Ciudad de México 04510, Mexico

**Keywords:** *Piper auritum*, Cytochrome P450, Ames test, safrole, heterocyclic amines, antimutagen

## Abstract

An ethanol extract of *Piper auritum* leaves (PAEE) inhibits the mutagenic effect of three food-borne aromatic amines (2-amino-1-methyl-6-phenylimidazo[4,5-*b*]pyridine (PhIP); 2-amino-3,8-dimethylimidazo[4,5-*f*]quinoxaline (MeIQx); 2-amino-3,4,8-trimethylimidazo[4,5-f]quinoxaline (4,8-DiMeIQx)) in the TA98 *Salmonella typhimurium* strain. Preincubation with MeIQx demonstrated in mutagenesis experiments that inhibition of Cytochrome P450 (CYP), as well as direct interaction between component(s) of the plant extract with mutagens, might account for the antimutagenic observed effect. Gas chromatography/mass spectrometry analysis revealed that safrole (50.7%), α-copaene (7.7%), caryophyllene (7.2%), β-pinene (4.2%), γ-terpinene (4.1%), and pentadecane (4.1%) as the main components (PAEE). Piper extract and safrole were able to inhibit the rat liver microsomal CYP1A1 activity that participates in the amines metabolism, leading to the formation of the ultimate mutagenic/ molecules. According to this, safrole and PAEE-inhibited MeIQx mutagenicity but not that of the direct mutagen 2-nitrofluorene. No mutagenicity of plant extract or safrole was detected. This study shows that PAEE and its main component safrole are associated with the inhibition of heterocyclic amines activation due in part to the inhibition of CYP1A subfamily activity.

## Introduction

Since ancient times, plants have been at the forefront of traditional medicine. Numerous epidemiological studies have reported the health benefits imparted by the consumption of certain fruits, vegetables, and their derived products. Indeed, most anti-cancer drugs in clinical use are naturally occurring compounds or their derivatives. Dietary factors can substantially influence cancer risk in humans [1]. Epidemiological studies pointed out the importance of diet in the development of cancer in human populations, suggesting that consumption of certain fruits, tea, coffee, vegetables, and red wine influence cancer risk in humans by conferring health benefits [2]. Plant metabolites like polyphenols and flavonoids have antioxidant and antiproliferative properties and can be found in such foods.

*Piper auritum* Kunth (hoja santa) is a bush that belongs to the Piperaceae family [3]. In Central America and México, it has been used as an analgesic, anti-inflammatory as well as hypoglycemic in traditional medicine [4]. These uses are supported by scientific studies in which its hypoglycemic, insulin resistance effect, hypolipidemic, and antioxidant activity have been reported [5,6]. Additionally, *P. auritum* is used as an antipyretic and diuretic for angina, gout, venereal diseases, colic, erysipelas, and headache as well as a local anesthetic, appetite stimulant, and wound poultice [7]. *Piper auritum* leaves are widely used in the south part of México as an important ingredient of traditional dishes, the heart-shaped leaves are used to wrap meat, fish, and tamales before cooking; chopped leaves are incorporated in stews and sauces and are cut into fine strips to be used as a condiment for soups and egg dishes.

The chemical composition of *P. auritum* aerial parts reveals the presence of phenylpropanoids as the main components, being safrole as the most dominant component [8]. Nevertheless, the safrole content in hoja santa essential oils from several parts of the world varies from 93% (Colombia and Costa Rica) to 40%–70% (México and Panama). Other components with anti-inflammatory, antioxidant, and antimicrobial activities including α-copaene, caryophyllene, γ-terpinene, and myristicin have been previously reported to be present in *P. auritum* [9].

On the other hand, heterocyclic aromatic amines (HAAs) are formed when fish, pork, chicken, or beef meat are exposed to high cooking temperatures [10]; these molecules have been tightly associated with the development of several types of cancer, specifically, a high intake of red meat is associated with colorectal tumors. After ingestion, HAAs can be bioactivated by the isoforms CYP1A1 and CYP1A2 of the cytochrome P450 system (Phase I). These enzymes perform an N-hydroxylation of the HAAs exocyclic amine group, which can be further esterified by Phase II enzymes acetyltransferases (NATs) or sulfotransferases (SULTs) [11]. The metabolic products of these reactions can spontaneously suffer heterolytic cleavage and be converted to electrophilic nitrenium ions with the potential to form covalent adducts with DNA, inducing genetic mutations that may lead to cancer initiation [12].

Modulation of xenobiotic metabolizing enzymes [13] is an approach in cancer prevention since one of the first steps related to the carcinogenesis process is the bioactivation of promutagens [14]. Here, we described the chemical composition and antimutagenic effect against food-borne aromatic amines of an ethanol extract from the leaves of Hoja santa and its main component; additionally, we explore their capacity to inhibit the activity of cytochrome P4501A subfamily involved in the metabolic activation of several environmental procarcinogens.

## Materials and methods

DMSO (Merck, Darmstadt, Germany); 7-ethoxyresorufin, resorufin, nitrofluorene, β-naphtoflavone, γ-terpinene, caryophylene, pentadecane, benzo[a]pyrene, 2-nitrofluorene (2NF), 4-nitroquinoline N-oxide (4-NQO), NADPH (Sigma Aldrich, St. Louis, MO, USA); α-copaene (Cayman Chemical Company, Ann Arbor, MI, USA); phenobarbital (Abbot Laboratories de México, S.A. de C.V.); safrole (US Biological Life Sciences, Swampscott, MA, USA). Supplies for the preparation of bacterial culture media were purchased from Becton Dickinson (Estado de México, México), and Oxoid (Lenexa, KS, USA). 2-Amino-3,4,8-dimethylimidazo[4,5-f]quinoxaline (DiMeIQx) was acquired from Santa Cruz Biotechnology, Dallas, TX, USA. 2-Amino-1-metil-6-fenylimidazo[4,5-*b*]pyridine (PhIP) and 2-Amino-3,8-dimethylimidazo[4,5-f]quinoxaline (MeIQx) was purchased from United States Biological (Salem, MA, USA).

## Preparation of ethanol extract

We modified the method proposed by Ikken *et al.* [15]. *Piper auritum* leaves (49.95 g) were collected from a family farm located the south of México city, México (19°21' 26.62416" N, 99°9' 55.64232" W; 2258 masl.) minced in small pieces in a mortar containing 180 ml ethanol, smashed with a pestle, and the mixture was let to repose for 72 h at ambient temperature. After filtration, ethanol was evaporated to dryness under N2 and pressure. The residue was suspended in 19 ml DMSO and stored at 4°C until it was used in the Ames test and determinations of CYP1A1 and 1A2 inhibitory activities.

## Analysis by GC–MS of phytochemicals

The ethanolic extract of *P. auritum* resulted after the filtration step mentioned above was analyzed by gas chromatography coupled to mass spectrometry (GC–MS) on a PerkinElmer Clarus SQ8S (Perkin Elmer, Shelton, CT, USA) implemented with a 30 m × 0.25 mm × 0.25 mm HP-5M capillary column (J&W Agilent, Santa Clara, CA, USA). An initial temperature of 40°C maintained for 3 min; 20°C/min up to 300°C for 10 min. Helium was used as the carrier gas at 1ml/min, and the ionization energy was 70 eV. Major peaks were identified using the NIST database. In an additional experiment under the same conditions mentioned above, injection of different concentrations of pure safrole, caryophyllene, pentadecane, terpinene, and copaene was additionally performed to confirm the identity of major volatiles and calculate their concentration in PAEE.

## Liver microsomal preparation

Five male Wistar rats (weighting 200–250 g) were housed in polypropylene cages under a 12-h light/dark cycle in an animal care facility located at the Instituto de Investigaciones Biomédicas, UNAM, Ciudad de México, México. The animals were allowed unrestricted access to laboratory rodent chow and distilled water and were euthanized after 5 days of daily i.p. injections of sodium phenobarbital at doses of 60 mg/kg (days 1, 2, and 3) and 30 mg/kg (day 4). 80 mg/kg β-naphthoflavone (5,6-benzoflavone) was also administered on the third day. Liver S9 fractions were prepared according to Maron and Ames [16]. Briefly, the livers were pooled, minced into small pieces, and homogenized in 150 mM KCl (3 ml/g liver wet weight). After centrifugation at 9000 *g* for 10 min, the supernatants (S9 fraction) were recovered and centrifuged at 100,000 *g* for 60 min; the pellet was resuspended in an equal volume of 100 mM potassium phosphate buffer (pH 7.4) and 250 mM saccharose and centrifuged again under the same conditions. The microsomal fractions were finally resuspended in 100 mM potassium phosphate (pH 7.4), 1.0 mM EDTA, 0.1 mM DTT, and 20% (v/v) glycerol and stored at −70°C. All the solutions and glassware were kept at 4°C. The protein concentrations in the microsomal fractions were determined according to Bradford [17], divided into 200 μl aliquots, and stored at −70°C until use. The Ethical Committee for Animals (CICUAL) of the Instituto de Investigaciones Biomédicas, UNAM, México approved the animal experiments for the preparation of the liver microsomal fractions.

## *Salmonella* mutagenicity assay

The *Salmonella* mutagenicity plate incorporation test was carried out as previously described [16]. *Salmonella typhimurium* strain TA98 was kindly donated by Dr Takehiko Nohmi (National Institute of Health Sciences, Tokyo, Japan). Liver microsomal protein obtained from rats pretreated with phenobarbital and β-naphthoflavone was used as the metabolic activation system. To 2 ml molten top agar with traces of histidine and maintained liquid at 45°C were added in the following order: 100 μl of *S. typhimurium* TA98 overnight culture, 100 μl of the test compound (HAAs or safrole dissolved in DMSO or PAEE extract) and 500 μl of the metabolic activation system S9 mix when required (4 mM NADP, S9 fraction (0.1 ml/ml), 8 mM MgCl2, 33 mM KCl, 5 mM glucose-6-phosphate and phosphate buffer pH 7.4). The mixture was gently vortexed and placed in a Petri dish containing Vogel–Bonner minimal medium; after incubation for 48 h at 37°C, the number of revertant colonies (His+) was counted. The toxicity of the tested agents was assessed by observing the background bacterial growth in the minimal agar plates due to traces of histidine in the medium [18]. In the preincubation method, the mixture containing appropriate components as required [bacterial culture (0.1 ml), S9 mix or phosphate buffer (0.5 ml), MeIQx (40 ng), PAEE (5 mg)] was placed in an empty sterilized tube and incubated for 20 min at 37°C. The mixture was poured in 2 ml molten top agar with traces of histidine and placed in a Petri dish containing Vogel–Bonner minimal medium; after incubation for 48 h at 37°C, the number of revertant colonies (His+) was counted. Data obtained with the preincubation method were analyzed using ANOVA and the post hoc Tukey test. A logarithmic transformation was necessary to achieve homogeneity of variance verified by Levene’s test.

## CYP1A1 and CYP1A2 activities

The ability of PAEE or its components to inhibit CYP1A1 and CYP1A2 activities was determined by the alkoxyresorufin-*O*-dealkylation (AROD) assay [19]. The incubation mixture was prepared in a 96-well microplate by adding buffer (50 mM Tris-HCl, 25 mM MgCl2, pH 7.6), substrate (ethoxyresorufin 1.25 µM for CYP1A1 and methoxyresorufin 12.5 µM for CYP1A2), hepatic microsomal protein (80 µg/well), and PAEE or its components at desired concentrations. An incubation mixture with the vehicle DMSO was evaluated as a control (100% of CYP1A activity). The microplate was incubated at 37°C for 3 min. Then, the reaction was initiated by adding 2.5 mM NADPH. Fluorescence measurements were performed every 20 s for 40 min at excitation and emission wavelengths of 530 and 585 nm, respectively. CYP1A activity was calculated with a standard curve of resorufin (5–500 pmol/ml), and results were expressed as pmol/mg prot/min or as a percentage of activity of control. The percentage of AROD activity was calculated relative to that of the vehicle (DMSO), which was assumed as 100%. PAEE and safrole concentrations that produce 50% inhibition in AROD activity (IC50) were estimated by non-linear regression of [inhibitor] versus % AROD activity curves.

## Results

The antimutagenic effect of PAEE against the three HAAs is presented in Table 1. A substantial reduction in the number of revertant colonies induced by the three amines was noted at 5 mg/plate of PAEE, reaching a reduction of 93%, 71%, and 74% of the mutagenicity exerted by MeIQx, 4,8-DiMeIQx, and PhIP, respectively. The spontaneous reversion rate of TA98 strain was almost reached at 50 mg/plate. The bacterial background grown was not modified in plates containing the highest PAEE concentration, indicating the absence of toxicity. PAEE was not mutagenic in the TA98 strain of *Salmonella typhimurium* at the highest concentration used in this experiment (Table 2) nor antimutagenic against 2-nitrofluorene or 4-nitroquinoline-1-oxide (data not shown).

**Table 1.** Antimutagenic effect of *Piper auritum* Kunth ethanolic extract (PAEE) against heterocyclic amines in *Salmonella typhimurium* TA98 tester strain in the presence of metabolic activation.

PAEE (mg/plate)	*S. typhimurium* TA98 *his*^*+*^ revertants^a^		
	Heterocyclic amines + S9		
	MeIQx	4,8-DiMeIQx	PhIP
0.00	1821 ± 62	2779 ± 310	515 ± 23
5.00	122 ± 16	793 ± 46	134 ± 34
15.00	69 ± 15	306 ± 49	76 ± 5
25.00	50 ± 10	197 ± 15	67 ± 2
35.00	36 ± 4	139 ± 10	58 ± 2
50.00	35 ± 4	99 ± 27	54 ± 9

^a^Number of revertant colonies found in three replicate plates from one representative experiment. MeIQx (2-amino-3,8-dimethylimidazo[4,5-*f*]quinoxaline), 40 ng/plate; 4,8-DiMeIQx (2-amino-3,4,8-trimethylimidazo[4,5-*f*]quinoxaline), 10 ng/plate; PhIP (2-amino-1-metil-6-fenylimidazo[4,5-*b*]pyridine) 500 ng/plate. S9 fraction was obtained from Wistar rats induced with Phenobarbital/β-naphtoflavone. TA98 + S9 + DMSO spontaneous reversion: 68 ± 8.

**Table 2.** Mutagenicity of *Piper auritum* Kunth ethanolic extract (PAEE) in *Salmonella typhimurium* TA98 tester strain in the presence or absence of metabolic activation.

*S. typhimurium* TA98 *his*^*+*^ revertants ^a^		
PAEE (mg/plate)	−S9	+S9
0	27 ± 12	41 ± 2
0.003	39 ± 4	48 ± 6
0.03	30 ± 2	47 ± 8
0.3	37 ± 3	35 ± 2
3.0	30 ± 1	42 ± 6
30.0	32 ± 11	32 ± 5
*Positive control*	676 ± 104^b^	346 ± 41^c^

^a^Number of revertant colonies found in three replicate plates from one representative experiment.

^b^2- Nitrofluorene 5 µg/plate.

^c^Benzo[a]pyrene 10 µg/plate. S9 fraction was obtained from Wistar rats induced with Phenobarbital/β-naphtoflavone.

**Figure 1.**
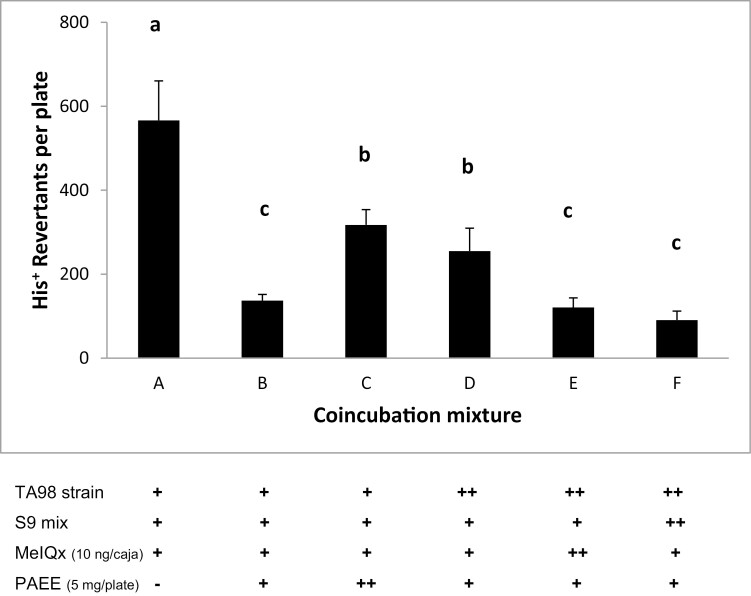
Antimutagenicity of *Piper auritum* Kunth extract against MeIQx in a coincubation experiment in *S. typhimurium* TA98 strain in the presence of metabolic activation. (**−**) not added; (+) added before preincubation; (**++**) added after 20 min. coincubation at 37°C. Similar letters above each column means no statistical difference; different letters mean statistic difference between treatments *P* < 0.001.

From the results shown above, MelQx was chosen to perform the *Salmonella* preincubation assay, and the results are presented in Fig. 1. Co-incubation mixture bar A represents the number of revertant colonies that resulted after exposure of TA98 strain to MeIQx plus metabolic activation, and bar B represents the antimutagenic action of PAEE. All combinations of components in the co-incubation mixtures assayed (C–F) reduced the number of mutant colonies. Furthermore, coincubation mixtures E and F resulted in the highest reduction in revertant colonies. Previous interaction in the preincubation mixture of PAEE with the S9 mix or MeIQx for 20 min caused a significant antimutagenic effect.

**Figure 2.**
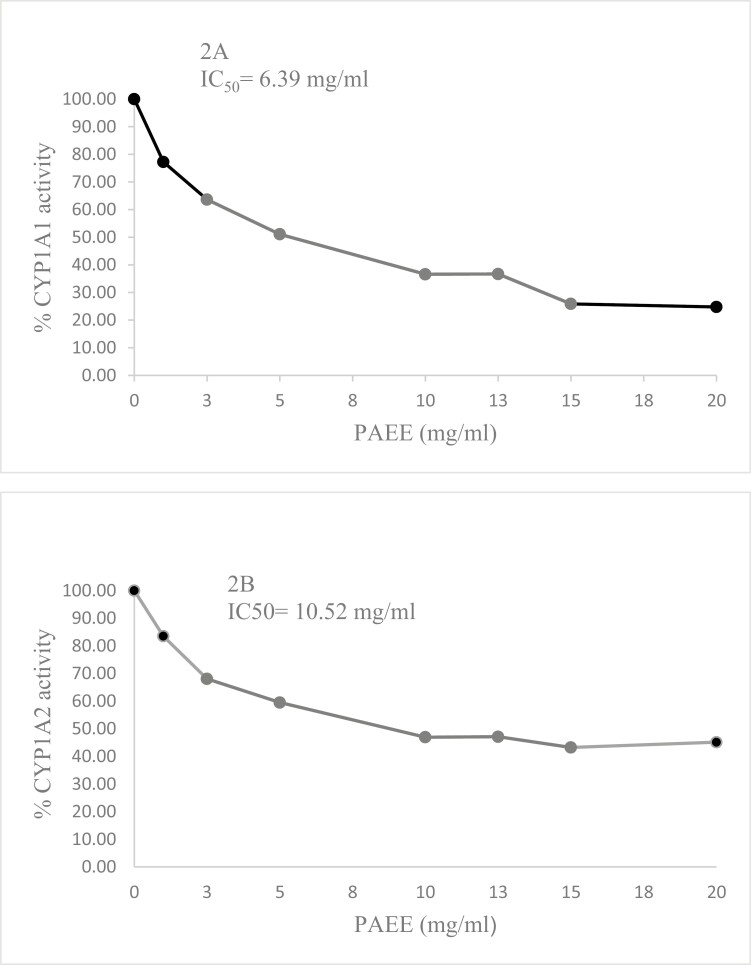
Inhibition of CYP1A1 (ethoxyresorufin-*O*-deethylase activity) (2A) and CYP1A2 (methoxyresorufin-*O*-demethylase activity) (2B) in rat liver microsomes by *Piper auritum* ethanolic extract. IC50 values were calculated by linear regression as described in the “Materials and methods” section.

Inhibition of CYP1A1 (ethoxyresorufin-*O*-deethylase activity) and CYP1A2 (methoxyresorufin-*O*-demethylase activity) in rat liver microsomes by PAEE is presented in Fig. 2. An inverse relationship between higher concentrations of PAEE and reduction in CYP1A1 and 1A2 activity was noted (Fig. 2a and Fig. b, respectively). The calculated 50% inhibitory concentration (IC50) of PAEE was 6.39 mg/ml for CYP1A1 and 10.52 mg/ml for CYP1A2.

Chemical composition of the main analytes in PAEE obtained by Gas chromatography/mass spectrometry method showed the following composition: safrole (50.7%), α-copaene (7.7%), caryophyllene (7.2%), β-pinene (4.2%), γ-terpinene (4.1%), and pentadecane (4.1%) (Table 3). Another 65 minor analytes were obtained for a 99.9% content. The concentration of the main five components of PAEE is presented in Table 4. Safrole (380.9 mg/l), caryophyllene (24.9 mg/l), α-copaene (22.36 mg/l), pentadecane (14.99 mg/l), and γ-terpinene (9.26 mg/l).

**Table 3.** Compounds (≥1%) obtained with ethanol from the leaves of *Piper auritum*.

Compounds	Retention time	% Peak área^a^
α-Pinene	5.756	2.68
β-Pinene	6.176	4.22
α- Terpinene	6.521	1.34
γ-Terpinene	6.889	4.09
α-Terpinolene	7.144	3.11
Safrole	8.779	50.71
α-Copaene	9.319	7.77
Caryophylene	9.642	7.25
Pentadecane	9.949	4.08
Germacrene	10.017	2.40
Patchoulene	10.092	1.03

^a^The values are the percentage of each peak concerning the total peaks identified by GC–MS. The table only includes peaks with an area higher than 1%.

**Table 4.** Concentration of the main chemical components in the *Piper auritum* ethanolic extract (PAEE).

Compounds	Concentration (mg/l)^*^	Retention time
γ-Terpinene	9.26	6.78
Safrole	380.90	8.65
α-Copaene	22.36	9.29
Caryophylene	24.91	9.61
Pentadecane	14.99	9.98

^*^PAEE was obtained as indicated in “Material and Methods” section.

The evaluation of the mutagenic and antimutagenic potential of safrole against MeIQx is shown in Tables 5 and 6, respectively. Safrole did not produce a dose–response mutagenic effect nor a duplication of the spontaneous reversion of the TA98 strain up to the highest concentration tested (Table 5). The bacterial background grown in the plates remained unaltered with respect to control plates without safrole. Instead, the presence of safrole reduced the number of revertants produced by MeIQx in a dose–response relationship, reaching a 64% inhibition at the highest concentration tested (Table 6). Only a 21% reduction in the mutagenicity exerted by the direct mutagen 2-nitrofluorene was noted. A combination of the main components of PAEE resulted in a 68.5% inhibition of MeIQx mutagenicity, contrasting with a 91% inhibition exerted by PAEE, but without safrole, only a 31.6% inhibition was reached. The addition of safrole at the concentration found in PAEE allows a decrease in mutagenicity of 72.5%, which means that this sole compound accounts for the major antimutagenic effect of PAEE (Table 7).

**Table 5.** Mutagenicity of safrole in *Salmonella typhimurium* TA98 tester strain in the presence or absence of metabolic activation.

*S. typhimurium* TA98 *his*^*+*^ revertants ^a^		
Safrole (µM)	−S9	+S9
0	29 ± 9	44 ± 6
30.82	35 ± 10	45 ± 6
154.1	40 ± 13	38 ± 8
308.2	40 ± 13	54 ± 17
1541	36 ± 10	51 ± 15
3082	36 ± 17	37 ± 14
*Positive control*	1059 ± 345^b^	518 ± 231^c^

^a^Mean number of revertant colonies ± standard deviation found in 6 replicated plates in two independent experiments (three plates/experiment).

^b^2-nitrofluorene 5 µg/plate.

^c^MeIQx 40ng/plate. S9 fraction was obtained from Wistar rats induced with phenobarbital/β-naphtoflavone.

**Table 6.** Antimutagenic effect of safrole in *Salmonella typhimurium* TA98 tester strain in the presence of metabolic activation.

Safrole (µM)	*S. typhimurium* TA98 *his*^*+*^ revertants^a^	
	Mutagens	
	2-NF −S9 (5 µg)	MeIQx + S9 (40 ng)
0.00	1501 ± 294	373 ± 75
30.82	1607 ± 169	396 ± 132
154.1	1502 ± 274	307 ± 88
308.2	1202 ± 171	229 ± 64
1541	1108 ± 140	160 ± 58
3082	1191 ± 320	133 ± 39

^a^Mean number of revertant colonies ± standard deviation found in nine replicated plates in three independent experiments (three plates/experiment). S9 fraction was obtained from Wistar rats induced with Phenobarbital/β-naphtoflavone.

**Table 7.** Antimutagenicity of main components in PAEE against MeIQx + S9 in the TA98 strain of *Salmonella typhimurium*.

Compounds	No. colonies/plate^a^	% Inhibition
MeIQx (40 ng/plate)	690 ± 80	0.0
PAEE (5 mg/plate)	61 ± 12	91.1
combination of analytes^b^	217 ± 54	68.5
combination of analytes w/o safrole	472 ± 32	31.6
safrole	190 ± 22	72.5
γ-Terpinene	652 ± 178	5.5
α-Copaene	639 ± 215	7.4
Caryophylene	639 ± 118	7.4
Pentadecane	648 ± 113	6.1

^a^Mean number of revertant colonies ± standard deviation found in six replicated plates in two independent experiments (three plates/experiment).

^b^Main compounds present in PAEE (5 mg): safrole (450.1 µM); terpinene (13.2 µM); copaene (21.0 µM); caryophylene (23.5 µM); and pentadecane (14.1 µM).

**Figure 3.**
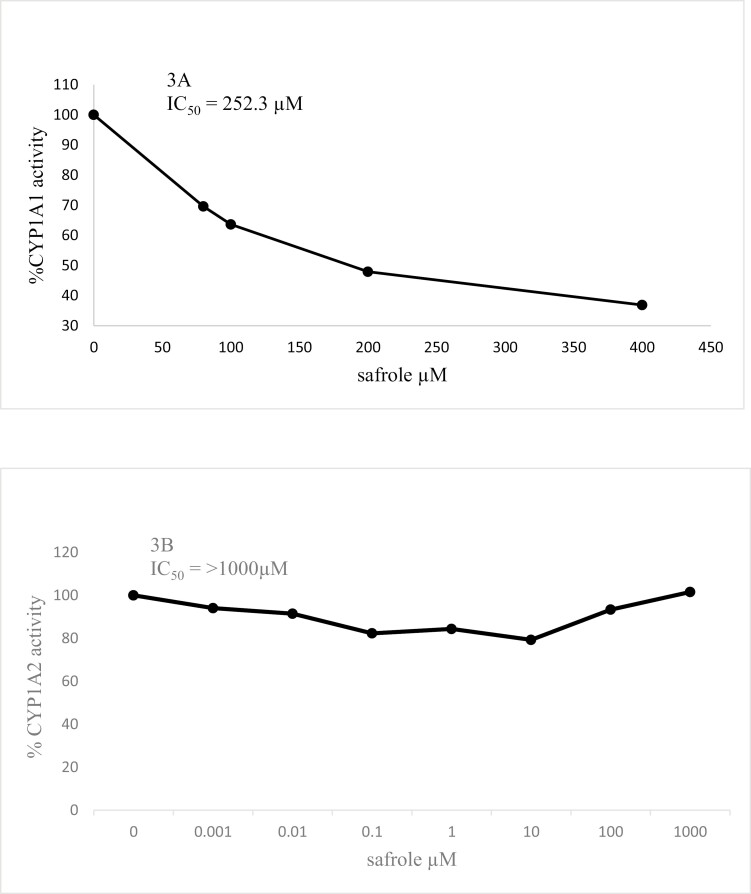
Inhibition of CYP1A1 (ethoxyresorufin-*O*-deethylase activity) (3A) and CYP1A2 (methoxyresorufin-*O*-demethylase activity) (3B) in rat liver microsomes by safrole. IC50 values were calculated by linear regression as described in the “Materials and methods” section.

The inhibition of CYP1A1 and 1A2 rat liver microsomal activities by safrole is shown in Fig. 3. Even when both cytochromes were inhibited, CYP1A1 was more sensitive than CYP1A2 to safrole’s inhibitory action. The IC50 for CYP1A1 was 252 µM, but the IC50 for CYP1A2 was not reached until safrole’s 1000 µM concentration. Furthermore, when a mixture of the main components was added to the incubation mixture at concentrations present in the plant extract, a 56% inhibition was observed (Table 8). In the presence of safrole alone, a similar inhibitory potency (58%) was reached, but when safrole was not in the mixture, only a 2.75% inhibition was observed (Table 8).

**Table 8.** Inhibition of CYP1A1 (ethoxyresorufin-*O*-deethylase activity) by PAEE main components.

Compounds	% CYP1A1 inhibition	CYP1A1 activity (pmol/mg prot/min)
No compound	0	455.04 ± 11.31
PAEE (5 mg/ml)	32.77	305.91 ± 0.71
Combination of analytes^a^	55.98	200.3 ± 4.19
Combination of analytes w/o safrole	2.75	442.51 ± 10.11
Safrole	58.22	190.13 ± 7.65
γ-Terpinene	0.71	451.8 ± 3.21
α-Copaene	0	463.45 ± 5.95
Caryophylene	0	466.09 ± 6.89
Pentadecane	0	469.38 ± 15.84

^a^Main compounds present in PAEE (5 mg): safrole (450.1 µM); terpinene (13.2 µM); copaene (21.0 µM); caryophylene (23.5 µM); and pentadecane (14.1 µM).

## Discussion

The presence of mutagenic substances in food is of great concern due to the high prevalence of cancer worldwide [20]. Concerning red meat and processed meat, since 2015, the IARC has cataloged them as probably carcinogenic and carcinogenic, respectively, to human beings [21]. Endogenous formation of nitrosamines from nitrite and amines in the acidic condition of the stomach as well as the presence of HAAs in meat cooked at high temperatures directly over the fire or over pan devices, promoted the development of stomach and colon cancer in animal models [22,23]. Furthermore, epidemiological evidence confirmed these results, leading to the search for strategies to avoid or counteract the deleterious damage provoked by exposition to these environmental mutagens/carcinogens. On the other hand, dietary factors negatively influence cancer development; plant metabolites like polyphenols and flavonoids possess antioxidant and antiproliferative properties and can be found in foods such as berries, citrus fruits, tea, and red wine. Fruits and vegetables that contain carotenoids and other antioxidants have been hypothesized to decrease lung cancer risks [24,25]. Edible plants are also a valuable source of antimutagens and have been considered a reliable option for chemoprevention [26].

Here, we report the antimutagenic potential of PAEE against three carcinogenic HAAs commonly found in cooked meat (Table 1).

We use the Ames preincubation method to get initial evidence to explain the mechanism of antimutagenicity of PAEE. A co-incubation mixture containing TA98 strain + S9 + MeIQx (Fig. 1 bar C) permits the formation of premutagenic lesions in the bacterial DNA and explores the capacity of PAEE to act as a bio-antimutagen through the modulation of DNA repair and replication mechanisms [27]. Co-incubation mixtures in D are designed to obtain evidence concerning the capacity of PAEE to interact with S9 or directly with MeIQx or both; meanwhile, E looks for a possible interaction of PAEE with S9. Finally, the co-incubation mixture in F explores the capacity of PAEE to form a molecular complex between some of its component(s) with MeIQx, impeding its activation by S9.

The results obtained with MeIQx in the Ames test preincubation assay (Fig. 1, co-incubation mixtures in E and F) suggest that the mechanisms involved could be the inhibition of the metabolic conversion of HAAs to their final electrophilic metabolites by CYP enzymes or a direct interaction of PAEE component(s) with MeIQx.

Guerrini *et al.* reported the antimutagenic property of two piper species (*Piper obliquum* and *Piper aduncum*) from Ecuador and suggested that the mechanism behind this effect could be the inhibition of CYP-mediated metabolism [28]. To corroborate this, we proceeded to test the potential of PAEE to inhibit CYP1A1/2 activity in rat liver microsomes (Fig. 2) and found that both isoforms of CYP1A subfamily are susceptible to inhibition by the complex vegetable mixture being CYP1A1 more vulnerable than CYP1A2. In the Ames test, we previously showed that recombinant human and rat CYP1A1 activate MeIQx and 4,8-DiMeIQx to mutagenic products [29]. Therefore, CYP inhibition may account to an essential degree for the antimutagenic effect exerted by PAEE.

We also tested the possible antimutagenic activity of PAEE against direct mutagens. The antimutagenic activity against 2-NF, and 4-NQO was scarce up to 30 mg of PAEE (data not shown).

Results from gas chromatography/mass spectrometry (Table 3) revealed that safrole was the main component in PAEE, followed by α-copaene, caryophyllene, β-pinene, γ-terpinene, and pentadecane. Safrole presented an evident antimutagenic activity against MeIQx in the Ames test TA98 strain in a dose–response relationship, reaching a 64% inhibition at the highest concentration tested (Table 6). Although the non-mutagenicity of safrole in the Ames test seen in this study agrees with other reports [28,30], as far as we know, the antimutagenic potential of this molecule has not been tested previously. Nevertheless, safrole is a substrate for CYP enzymes [31], leading to interference with other CYP substrates inhibiting their metabolism. Our results (Fig. 3) confirm this notion, which demonstrates the inhibition of CYP1A1, but not CYP1A2 by safrole. Our results partially follow Ueng [32], who reported that safrole is an inhibitor of human CYP1A2 and CYP1A1 with IC50 values of 5.7 µM and >100 µM, respectively. The apparent disagreement of these data with those reported here could be due to the different species of the CYP used. We used rat liver microsomes as CYP source in comparison with the human recombinant CYP isolated from *Escherichia coli* used by Ueng [32]

We demonstrate the importance of safrole in the antimutagenic as well as the CYP inhibitory properties of PAEE by experiments involving a mixture of its main components. Data in Tables 7 and 8 show that safrole accounts for more than half of the antimutagenicity and CYP inhibitory activity of PAEE, respectively. These results also demonstrate a lack of a visible interaction of safrole antimutagenic and CYP inhibitory properties with the rest of the main components tested.

Because the total CYP inhibitory and antimutagenic properties of PAEE cannot be explained by the presence of one of its components, we cannot rule out the importance of other molecules present in the complex mixture. Other green vegetable components reported to have antigenotoxic properties, like chlorophyl, were also investigated by us, but their antimutagenic effect was negligible (data not shown). With respect to caryophyllene, its CYP1A1 and CYP1A2 IC50 inhibitory concentration was higher than 10 000 µM (data not shown).

One of the first alkenylbenzenes contained in plant essential oils identified to have carcinogenic properties producing liver tumors in rodents was safrole [33]. Risk assessment of the consumption of plant food supplements (PFS) containing safrole based on the so-called margin of exposure concludes that the use of PFS containing safrole might raise a potential concern for human health [34]. However, it must be noted that this kind of analysis is based on studies administering high doses of pure safrole to rodents instead of dosing a form of multicomponent extract or the botanical as such. This is because results obtained with the herb or its extract containing the genotoxic compound in its food matrix are different from those obtained with the purified carcinogenic compound [35]. Such differences may arise when additional compounds in the food matrix interfere with the carcinogenic activation of the genotoxic compound. This is the case for estragole, which is present in plants such as fennel (*Foeniculum vulgare*) and basil (*Ocimum basilicum*). CYP and sulfotransferase enzymes transform estragole to 1'-hydroxyestragole and 1'-sulfooxyestragole, respectively, and the breakdown of this last metabolite releases the electrophilic carcinogenic compound [36]. A methanolic basil extract inhibits sulfotransferase activity impeding the bioactivation of 1'-hydroxyestragole to its final carcinogenic metabolite [37]; later, Alhusainy *et al.* [38] identified nevadensin as the inhibitory molecule.

As described above for estragole, safrole is first hydroxylated by CYP to 1'-hydroxysafrole, followed by sulfonation mediated by sulfotransferases, giving rise to the ultimate carcinogenic metabolite 1'-sulfooxysafrole that forms covalent adducts with DNA [33,39]. Morimitsu *et al.* [40]reported the flavonoids inhibition of sulfate conjugation in rat cultured hepatocytes and liver subcellular preparations. Furthermore, malabaricone C-containing mace extract inhibits sulfotransferase and prevents DNA adduct formation by safrole. These results indicated that the matrix effect by the presence of compounds that inhibit biotransformation and activation of genotoxic carcinogens in plant extracts must be taken into account to better estimate risk assessment.

Edible plants containing safrole are widely used by human beings as a food condiment and in traditional medicine; taking into account the rodent carcinogenicity of safrole, additional *in vivo* experiments must be carried out in order to estimate the risk derived from exposure to safrole-containing plants, considering dosing the botanical as such or within a multicomponent extract to evaluate the presence of other herbal ingredients compared to the exposure to safrole as a pure compound. Additionally, the consequences for human health of the findings reported here, must be further evaluated in the animal and epidemiological models to clarify if the intake of plant-containing *in vitro* antimutagenic agents, like safrole, may constitute a safe way to equilibrate the ingestion of genotoxic molecules already present in daily food intake or forming during its cooking.

## Supplementary data

Supplementary data is available at *Mutagenesis* online.

geae011_suppl_Supplementary_Data_S1

geae011_suppl_Supplementary_Data_S2

geae011_suppl_Supplementary_Data_S3

geae011_suppl_Supplementary_Data_S4

geae011_suppl_Supplementary_Data_S5

geae011_suppl_Supplementary_Data_S6

geae011_suppl_Supplementary_Data_S7

## Data Availability

The data underlying this article are available in the article.
