# Supplementary material for: Piper auritum ethanol extract is a potent antimutagen against food-borne aromatic amines: mechanisms of action and chemical composition
Source: Mutagenesis. 2024 Mar 23;39(6):301–9. doi: 10.1093/mutage/geae011 (PMC11529617; doi:10.1093/mutage/geae011)
Supplement: geae011_suppl_Supplementary_Data_S7 [file geae011_suppl_supplementary_data_s7.docx]

|  | Experiment 1 | | | Experiment 2 | | |
| --- | --- | --- | --- | --- | --- | --- |
| Treatment | Revertants | Mean | SD | Revertans | Mean | SD |
| TA98 | 44 | 42 | 7 | 29 | 36 | 8 |
|  | 35 |  |  | 45 |  |  |
|  | 48 |  |  | 33 |  |  |
| TA98+S9 | 33 | 36 | 6 | 28 | 44 | 15 |
|  | 32 |  |  | 47 |  |  |
|  | 43 |  |  | 57 |  |  |
| TA98+DMSO | 42 | 41 | 1 | 31 | 34 | 3 |
|  | 40 |  |  | 36 |  |  |
|  | 42 |  |  | 36 |  |  |
| TA98+S9+DMSO | 43 | 37 | 6 | 39 | 40 | 3 |
|  | 31 |  |  | 44 |  |  |
|  | 36 |  |  | 38 |  |  |
| Control -S9 (5 μg 2NF/plate) | 1900 | 1977 | 234 | 2124 | 2056 | 64 |
|  | 2240 |  |  | 1996 |  |  |
|  | 1792 |  |  | 2048 |  |  |
| Control +S9  (40 ng MeIQx/plate) | 650 | 727 | 106 | 668 | 652 | 27 |
|  | 848 |  |  | 667 |  |  |
|  | 684 |  |  | 621 |  |  |
| PAEE 5 mg/mL+S9  (40 ng MeIQx/plate) | 62 | 54 | 12 | 77 | 67 | 10 |
|  | 60 |  |  | 66 |  |  |
|  | 41 |  |  | 57 |  |  |
| Mixture of analytes+S9  (40 ng MeIQx/plate) | 198 | 176 | 21 | 304 | 257 | 44 |
|  | 157 |  |  | 252 |  |  |
|  | 173 |  |  | 216 |  |  |
| Mixture of analytes -safrol+S9  (40 ng MeIQx/plate) | 452 | 469 | 48 | 464 | 474 | 15 |
|  | 524 |  |  | 491 |  |  |
|  | 432 |  |  | 466 |  |  |
| Safrol 0.731 mg/mL+S9  (40 ng MeIQx/plate) | 206 | 193 | 14 | 152 | 188 | 31 |
|  | 178 |  |  | 210 |  |  |
|  | 194 |  |  | 202 |  |  |
| Terpinene 0.0018 mg/mL+S9  (40 ng MeIQx/plate) | 840 | 763 | 165 | 530 | 541 | 122 |
|  | 876 |  |  | 425 |  |  |
|  | 574 |  |  | 669 |  |  |
| Copaene 0.0043 mg/mL+S9  (40 ng MeIQx/plate) | 630 | 721 | 289 | 543 | 557 | 109 |
|  | 488 |  |  | 672 |  |  |
|  | 1044 |  |  | 456 |  |  |
| Caryophylene 0.0048+S9  (40 ng MeIQx/plate) mg/mL | 770 | 701 | 73 | 434 | 577 | 133 |
|  | 708 |  |  | 599 |  |  |
|  | 624 |  |  | 697 |  |  |
| Pentadecane 0.003 mg/mL+S9  (40 ng MeIQx/plate) | 732 | 732 | 24 | 678 | 564 | 101 |
|  | 756 |  |  | 486 |  |  |
|  | 709 |  |  | 527 |  |  |

Data set of *Salmonella* experiments reported in Table 7.
